# Mucopolysaccharidosis Type II: One Hundred Years of Research, Diagnosis, and Treatment

**DOI:** 10.3390/ijms21041258

**Published:** 2020-02-13

**Authors:** Francesca D’Avanzo, Laura Rigon, Alessandra Zanetti, Rosella Tomanin

**Affiliations:** 1 Laboratory of Diagnosis and Therapy of Lysosomal Disorders, Department of Women’s and Children ‘s Health, University of Padova, Via Giustiniani 3, 35128 Padova, Italy; frale100@gmail.com (F.D.); alessandra.zanetti@unipd.it (A.Z.); 2 Fondazione Istituto di Ricerca Pediatrica “Città della Speranza”, Corso Stati Uniti 4, 35127 Padova, Italy; laura.rigon@unipd.it; 3 Molecular Developmental Biology, Life & Medical Science Institute (LIMES), University of Bonn, 53115 Bonn, Germany

**Keywords:** Mucopolysaccharidosis type II, Hunter syndrome, lysosomal storage disorders, X-linked trait, glycosaminoglycans, diagnosis, therapy, pathogenesis, animal model

## Abstract

Mucopolysaccharidosis type II (MPS II, Hunter syndrome) was first described by Dr. Charles Hunter in 1917. Since then, about one hundred years have passed and Hunter syndrome, although at first neglected for a few decades and afterwards mistaken for a long time for the similar disorder Hurler syndrome, has been clearly distinguished as a specific disease since 1978, when the distinct genetic causes of the two disorders were finally identified. MPS II is a rare genetic disorder, recently described as presenting an incidence rate ranging from 0.38 to 1.09 per 100,000 live male births, and it is the only X-linked-inherited mucopolysaccharidosis. The complex disease is due to a deficit of the lysosomal hydrolase iduronate 2-sulphatase, which is a crucial enzyme in the stepwise degradation of heparan and dermatan sulphate. This contributes to a heavy clinical phenotype involving most organ-systems, including the brain, in at least two-thirds of cases. In this review, we will summarize the history of the disease during this century through clinical and laboratory evaluations that allowed its definition, its correct diagnosis, a partial comprehension of its pathogenesis, and the proposition of therapeutic protocols. We will also highlight the main open issues related to the possible inclusion of MPS II in newborn screenings, the comprehension of brain pathogenesis, and treatment of the neurological compartment.

## 1. Introduction

Mucopolysaccharidosis type II (MPS II, MIM # 309900), also known as Hunter syndrome, is a rare genetic disorder that is inherited as an X-linked trait, with an incidence rate ranging from 0.38 per 100,000 live newborns in Brazil to 1.09 per 100,000 live newborns in Portugal. European countries generally present a lower incidence than East Asian countries, where, in some of them, MPS II incidence accounts for about 50% of all mucopolysaccharidoses (MPSs) [1]. MPS II belongs to the group of lysosomal storage disorders (LSDs) and is due to a deficit of the lysosomal enzyme iduronate 2-sulphatase, which catalyzes the hydrolysis of 2-sulphate groups of dermatan sulphate (DS) and heparan sulphate (HS). Therefore, its deficit causes the pathological accumulation of these two glycosaminoglycans (GAGs) and dysfunction of most organ-systems, including the brain, in the majority of patients, thus representing a severe clinical phenotype [2].

## 2. History

**Figure 1:**
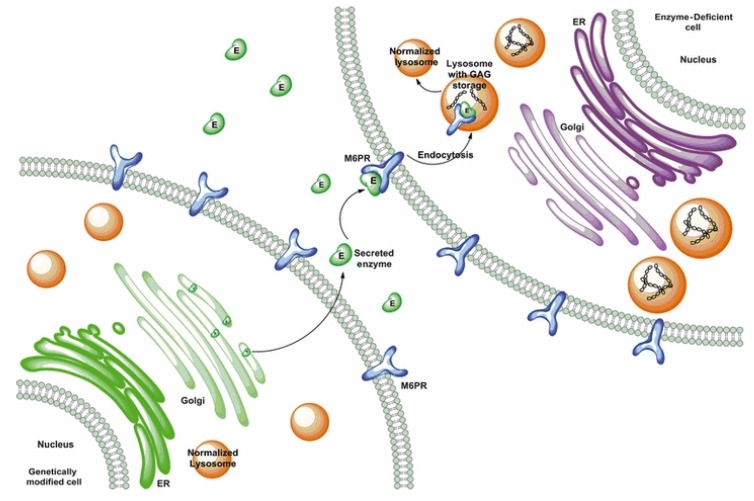
Cross-correction. Lysosomal enzyme trafficking and correction of adjacent cells. Obtained from Tomanin et al. 2012 [21], with permission of John Wiley and Sons publisher.

The syndrome was first described by the Canadian physician Charles Hunter in 1917 [3], following the clinical observation of two brothers, which was later recognized as MPS II. However, until 1952, nobody ever hypothesized the biochemical cause of the disease [4]. Two years later, Gertrud Hurler described other two cases with clinical similarity to that described by Hunter [5]. These two additional cases were long interpreted as being affected by the same disease previously reported, although these subjects also presented with gibbus and important corneal clouding. The Hurler and Hunter syndromes were still considered as one disease in 1968—thus half a century later—when a paper by Constantopoulos [6] defined the disease as the Hunter–Hurler syndrome. In the same year, Fratantoni and colleagues postulated that Hunter syndrome could be an X-linked clinically less severe variant of Hurler syndrome [7]. Finally, in 1978, a publication by Lorincz described the distinct pathogenesis of the two diseases [8], thus making them distinguished disorders. From a biochemical point of view, it was only in 1968 that scientists started to hypothesize that these two diseases were caused by the lack of mucopolysaccharide breakdown [7], and in the same year, the same group of scientists observed in vitro the phenomenon of cross-correction, by co-culturing Hunter and Hurler fibroblasts, through which they could observe a reciprocal correction [9] (Figure 1). This allowed them to hypothesize the existence of diffusing factors passing from one cell to another and helping to correct the pathological phenotype. This last paper represents a real milestone in the history of Hunter and Hurler diseases as it was the fundamental basis from which enzyme replacement therapy (ERT) developed. Two years later, the same research team, headed by Elizabeth Neufeld, proposed that this diffusing factor, deficient in Hunter syndrome, might be a protein [10], whose isolation and characterization was conducted by this same group in 1972 [11]. In the meantime, other so-called “corrective factors” were suspected to be associated with other LSDs, including Hurler disease (MPS I) and Sanfilippo syndrome (MPS III) [12]. In 1973, the same group of researchers, by analyzing several in vitro experiments that they had progressively conducted and putting together different lines of evidence, were able to conclude that the Hunter corrective factor was a sulphatase, acting on the sulphated residues of iduronic acid [13]. The human gene was first isolated in 1990, when the cDNA was sequenced [14]. Sequencing of the gene with definitions of coding sequences, intron boundaries, and the 5′ promoter region was completed in 1993 [15,16]. Then, it was only in 1995 that the existence of a pseudogene next to the functional gene was first described [17], and in the same year, scientists started to suspect that its presence so close to the gene might give rise to phenomena of homologous recombination with the *IDS* gene, thus representing a cause of pathology [18].

Due to several features, Hunter disease is an interesting pathology, and it represents a good model of study for disorders with a genetic origin: it is a monogenic disorder, it is due to alterations of a housekeeping gene, both the gene and the deriving protein are well-known, and a mouse model of the disease has been available for twenty years [19]. Moreover, ERT for the disease has been available since 2006 and, for what is known, the levels of administered protein are not critical. Therefore, gene therapy protocols can also be investigated and applied for treatment, with different gene expression levels being well-tolerated [20].

## 3. Molecular Basis

Mucopolysaccharidosis type II is a genetic X-linked recessive disorder. The *IDS* gene (HGNC ID:5389; ENSG00000010404) maps at the chromosomal region Xq28, spans 44 kb, and is structured in nine exons.

**Figure 2:**
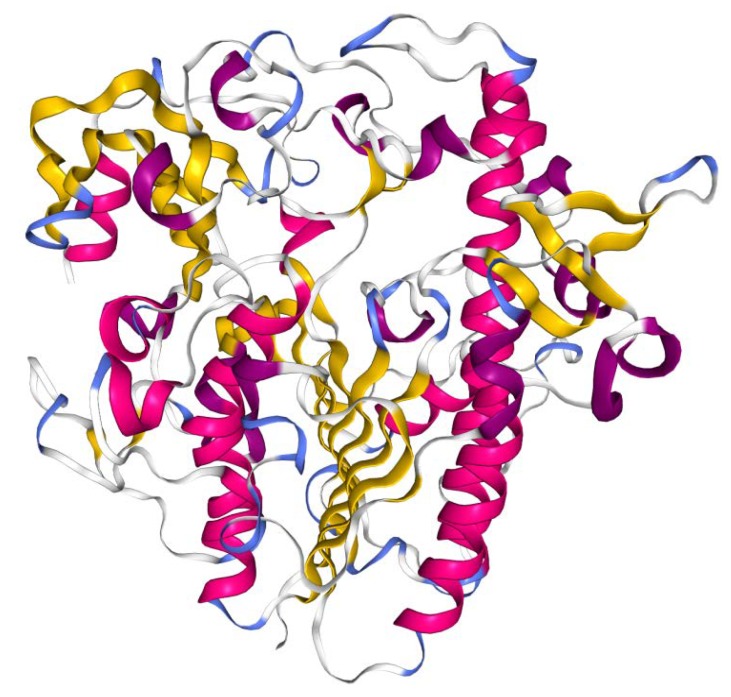
Crystal structure of IDS protein. Cartoon diagram colored by secondary structure. Image from the RCSB PDB (http://www.rcsb.org/) [35] of PDB ID 5FQL [23], created with NGL viewer [36].

The gene encodes for a 550 amino acid polypeptide which is processed into a mature protein. The crystal structure of recombinant clinical-grade 76 kDa glycosylated IDS evidenced that the mature form of the enzyme is monomeric and is composed of two subdomains. The N-terminal subdomain SD1 (amino acid 34–443), previously reported as the 42 kDa ‘heavy’ chain [22], contains the catalytic core and remains stably associated with the subdomain SD2 (residues 455-550, reported as 14 kDa ‘light’ chain), forming a large hydrophobic packing interface [23] (Figure 2). Cysteine 84 is post-translationally modified to formyl-glycine, which is a key catalytic residue of the active site. The IDS enzyme (EC:3.1.6.13) catalyses hydrolysis of the C2-sulphate ester bond of 2-O-sulfo-α-L-iduronic acid residues in DS and HS [23].

In the early 90s, the identification of patients carrying heterozygous variants in exon 3 by genomic PCR [17] suggested the presence of a locus homologous to *IDS*. Its characterization evidenced a pseudogene called *IDSP1* (HGNC ID:5389) located 3.9 kb from *IDS* on the telomeric side and in the opposite orientation. It includes sequences homologous to exons 2 and 3 and introns 2, 3, and 7, with exon 3 showing 100% sequence identity [24].

To date, 658 variants have been reported in the *IDS* gene (HGMD professional 2019.1). Almost half of them are missense/nonsense mutations, followed by small deletions, splicing variants, gross deletions, complex rearrangements, small indels, and gross insertions (Table 1). Most reported complex rearrangements are homologous intrachromosomal recombinations between *IDS* and its pseudogene *IDSP1*, generally leading to inversion of the genomic region between intron 7 of *IDS* and its homologous region of *IDSP1*, without any appreciable deletions or insertions [18,25,26,27]. However, in some cases, the recombinational events are associated with deletions of the *IDS* fragment involved in recombination and with insertion of part of the pseudogene [27,28,29,30,31]. Moreover, in a few cases, wide deletions, not associated with homologous recombination, involving the whole or part of the *IDS* gene, and extending to contiguous genes, have been described [29,32,33,34].

Although MPS II is an X-linked disorder, rare sporadic cases in females have been reported, most of which were caused by non-random X chromosome inactivation (XCI), commonly called skewed X-inactivation. Most described cases carried the mutated maternal allele, while only a few cases carried de novo mutations. No preferential types of mutations have been detected in Hunter females since missense, nonsense, synonymous, large deletions, indels, or even chromosomal translocation have been equally reported [31,37,38,39,40,41,42,43]. Interestingly, a case of a Hunter female giving birth to a healthy girl has also been described [38].

Hunter syndrome is characterized by a high genetic heterogeneity, as no highly recurring mutations have been reported so far, although some variants seem to be slightly more frequent [44]. As a consequence, genotype–phenotype correlations are difficult to investigate for most types of variants, although, as Hunter syndrome is an X-linked trait, each patient phenotype is due to the expression of a single variant. Only large deletions/insertions, complex rearrangements, and nonsense and splicing variants are commonly associated with severe forms [45,46]. 

The description of different phenotypes in subjects carrying the same variants likely implies the involvement of other, not yet clarified, genetic modifying processes or environmental factors determining the phenotype [44,47,48].

## 4. Clinical Features and Degrees of Severity

As *IDS* is a housekeeping gene, MPS II patients may be affected in most organ-systems, to different degrees, and a considerable heterogeneity in disease presentation needs to be taken into account [49]. Patients present with an altered level of urinary GAGs (uGAGs), while shared clinical signs and symptoms include coarse facial features, skeletal deformities and joint stiffness, growth retardation with a short stature, respiratory and cardiac impairment, including a diffuse valvulopathy [50], inguinal and umbilical hernias [51], organomegaly (mainly enlargement of the liver and spleen), and neurological involvement in at least two-thirds of cases [49,52,53]. Patients also present ENT (ear, nose, and throat) manifestations (hearing loss, adeno-tonsillar hypertrophy, and frequent ear and upper respiratory infections), sleep disturbances and obstructive apnea [54], and retinal deterioration [2]. Among major clinical alterations, cardiac-respiratory failure is commonly the cause of death [55], which occurs before adulthood for severe forms, while those with mild forms can survive until late adulthood.

For clinical purposes, Hunter syndrome has long been conventionally described as presenting two main forms—the attenuated and the severe one—although it appears clear that a continuum of different forms can be observed. Patients usually appear normal at birth, and somatic signs commonly start between 2 and 4 years of age, although severe forms generally present earlier. The attenuated forms may present with a slow progression of peripheral signs/symptoms, absent or reduced cognitive problems, and no behavioral difficulties. The main distinction between the two wide classes of forms is related to the presence/absence of neurological involvement, mainly represented by cognitive impairment and severe behavioral problems [52]. Together with this criterion, distinction between the two forms is sometimes also associated with the presence/absence of progression of the brain involvement [51]. On this basis, patients presenting some cognitive problems, although not showing a clear progression, or patients showing late CNS involvement, may be at first classified as ‘mild’ or ‘attenuated’ [51]. A notable distinction has to be maintained between cognitive/behavioral and general neurological problems, which do not have to be confused, since general neurological problems, including, for example, spinal cord compression, may be equally identified in the majority of patients, although in some cases, they do not influence their cognitive abilities [56].

Possibly due to the rarity of the disease and the very high number of different and private genetic variants reported for the *IDS* gene, so far, a genotype–phenotype correlation has never been clearly stated, with some exceptions, as previously discussed. Therefore, presently, we cannot assess whether attenuated phenotypes are associated with some residual enzymatic activity, and the pathogenic reasons underlying the existence of different forms, with or without neurological impairment, remain an open issue.

### Female Carriers

Several studies have shown that most MPS II carriers are asymptomatic, although presenting, in most cases, slightly lower plasma and leukocyte IDS activities with respect to non-carriers, with values that often overlap with normal ones [57,58]. In only one study performed on a small group of carriers, the presence of some mild clinical manifestations typical of MPS II was evidenced in three women; however, these women showed a moderately skewed XCI pattern [59]. The absence of symptoms in MPS II carriers with respect to Fabry carriers (another X-linked lysosomal storage disorder), who are often symptomatic, has been hypothesized to be the consequence of a different efficiency of cross-correction by the functional enzyme secreted by the cells expressing the non-mutated gene [57,60].

## 5. Diagnosis

Clinical diagnosis of MPS II is not so straightforward as it relies on the recognition of signs and symptoms that are often not specific for MPS II, but can be shared with other LSDs or other non-lysosomal disorders. However, in most cases, the first suspicion of MPS II derives from the typical facial features of the patients, especially in the most severe ones. 

When a suspicion of MPS II is formulated, the next step is to evaluate whether uGAG excretion is increased through the quantitation of GAG levels in 24-hour urine samples [61,62]. This should be followed by a qualitative analysis performed by electrophoresis [63,64] or mass spectrometry [65], in order to identify the GAG species preferentially accumulated. It should be noted that false positive/negative results can be produced by both quantitative and qualitative methods; indeed, alterations of the GAG composition can be due to urine contamination by blood, the concomitant administration of specific drugs, or the presence of other diseases [66,67].

Once it is established that uGAGs are quantitatively and qualitatively altered, the second step is to measure the enzymatic activity. As the IDS protein is present in all cells, except for mature red blood cells, activity could be evaluated in different cells or bodily fluids, such as cultured skin fibroblasts, leucocytes, plasma, and serum. The enzymatic activities of one or more sulphatases are usually tested together with IDS activity so as to exclude multiple sulphatase deficiency (MIM #: 272200) [2]. Most Hunter patients present no residual IDS activity [68], whilst some attenuated patients have been reported to have 0.2%–2.4% of a healthy control’s activity [69]. A recent paper reported no correlation between residual IDS activity and patients’ phenotypes, and just one patient with an attenuated form showed a residual activity of 14.6% [45]. Molecular genetic testing allows the identification of the disease-causing genetic variant, hence confirming the results of the biochemical evaluations. Molecular analysis is generally carried out by the PCR amplification of *IDS* exons and their 3′ and 5′ boundaries, followed by Sanger sequencing. It should be noted that a strategy for discriminating exon 3 of *IDS* from the homologous region of *IDSP1* should be included in the course of the analysis. With only one allele being amplified in male patients, partial or whole gene deletions are also detected by simple PCR amplification. If negative results are obtained by this approach, further investigations need to be implemented to identify potential recombinations between the homologous regions of the *IDS* gene and its pseudogene. This can be performed through the simple PCR-based method of analysis of recombinants described by Lualdi [70], which allows the characterization of different types of recombinants, also permitting an analysis of the carrier status of the patient’s female relatives.

In rare cases, additional molecular analyses are needed, such as mRNA analysis, to evidence the effect of a putative splicing variant on mRNA processing [47], or array CGH (comparative genomic hybridization) analysis, to detect large deletions and/or duplications involving the *IDS* gene [71]. In the last years, the development of next generation sequencing (NGS) techniques has allowed the diagnosis of several cases of MPS II by the application of multi-gene targeted panels or whole exome sequencing [72,73]. 

The identification of heterozygous carriers is usually performed by genetic analysis, as the measurement of both IDS activity and uGAGs is not reliable [60].

### 5.1. Differential Diagnosis

Differential diagnosis for Hunter syndrome should include all the other mucopolysaccharidoses and lysosomal storage disorders with overlapping signs and symptoms, such as mucolipidosis II alfa/beta, III alfa/beta, and III gamma, mannosidosis, fucosidosis, and multiple sulphatase deficiency. Additionally, other non-lysosomal conditions associated with macrocephaly and/or organomegaly and presenting with developmental delay should be considered in the diagnostic process. Qualitative uGAG analysis guides the clinician through the different MPSs, whilst not discriminating between MPS I and MPS II. Only an evaluation of enzyme activities followed by genetic analysis can lead to a definite diagnosis [2,68].

### 5.2. Prenatal Diagnosis

Prenatal analysis should be performed in fetuses at risk of MPS II. As MPS II is an X-linked disorder, generally, at first, the fetal sex is determined, and then, in the case of a male karyotype, biochemical evaluations are performed. Enzymatic evaluations can be performed on both fresh and cultured chorionic villus samples or on cultured amniotic fluid cells. If the genotype of the family index case is known, a molecular genetic test can be carried out. The application of both enzymatic and genetic tests increases the reliability of the diagnosis [52,64].

### 5.3. Newborn Screening (NBS) 

The incidence of Hunter syndrome, one of the most common mucopolysaccharidoses, the availability of an ERT since 2006, and the clear evidence that its early application provides a better clinical efficacy, have sustained, for a while, the request to include the IDS enzyme activity analysis within commonly applied newborn screenings (NBSs) [74,75].

In the last ten years, many pilot NBS programs for LSDs have been carried out in several developed countries using, in most cases, tandem mass spectrometry (MS/MS) or fluorometry techniques for a direct measurement of enzymatic activity in dried blood spots (DBSs) [76]. Only a few of these studies have included MPS II among the disorders tested [77,78,79,80,81,82] and a few of them are developing from pilot studies to population programs. Indeed, a population-based screening for MPS I, MPS II, and MPS VI was recently completed in Taiwan by MS-MS [83]. In this program, more than 130,000 infants were evaluated and three newborns carrying pathogenic variants in the *IDS* gene were identified. Similarly, in Illinois (USA), a population screening involving more than 160,000 infants was carried out, leading to the identification of one case of MPS II [84]. 

In addition, other approaches based on the measurement of GAGs in urine and blood samples through liquid chromatography-MS/MS have been developed in order to be potentially used as newborn screening tools for MPSs [85,86], also in combination with enzyme activity assays [87].

However, as some NBSs have become whole-population programs, several ethical issues associated with these programs are still widely debated [88]. In fact, the vast majority of MPS patients and families support NBSs, even in the absence of specific treatments [89,90], due to the possibility of obtaining an early diagnosis for their kids; however, the possible identification of genomic variants of unknown significance (VUS), pseudodeficiency alleles, or late-onset forms, surely represents a cause of notable distress and anxiety for the families, given the clinical severity of the diseases that their kids could potentially develop. These ethical issues should be carefully considered, and the application of expanded screening programs should include a long-term follow-up of subjects with an abnormal NBS result [91]. Furthermore, from this perspective, screening programs should always plan the inclusion of specific professional figures in association with clinical geneticists and genetic counsellors, as psychologists and psychotherapists, for helping families to handle such a difficult situation.

## 6. Treatment

Historically, the management of MPS II has been palliative and focused on the treatment of signs and symptoms. Since the discovery of the biochemical and genetic bases of the disease, which occurred in the 1970s and 1990s, respectively, many studies have been performed, exploiting different strategies, with the aim of developing a specific therapy for the disease. These efforts led to the introduction in clinical practice of hematopoietic stem cell transplantation (HSCT) in the 1980s and of enzyme replacement therapy (ERT) in 2006. Although these therapeutic strategies, primarily ERT, are nowadays used as therapeutic options for the treatment of MPS II, many questions remain about the efficacy and safety of their application, leaving this research field open.

In the following paragraphs, the different therapeutic strategies for MPS II, both in use and in the phase of development, will be described.

### 6.1. Management of Symptoms

Symptomatic therapies have long been used to relieve general symptoms of the disease, many of which have remained unsolved following the availability of more targeted treatments, such as ERT. In addition, the disease is so complex, involving many districts, that management is typically challenging and requires a multidisciplinary approach [92].

Since common therapeutic interventions do not reach the brain compartment, symptoms related to neurological involvement have so far remained mainly uncured and can only be treated symptomatically. Among these are communicating hydrocephalus and spinal cord compression, commonly treated with surgery for decompression, and seizure, mainly affecting severe patients and treated with anti-convulsant drugs. Patients also suffer from a lack of sleep, which might contribute to behavioral problems [92,93]. Carpal tunnel syndrome, a neuropathy rarely seen in kids, is instead more common in Hunter children and often requires surgery intervention [92]. Moreover, hearing support for deafness, or in some cases myringotomy with the placement of ventilating tubes, is commonly required [92,94], as well as eye surgery, to correct retinopathy or corneal opacity, although this last example is not a prominent sign of MPS II [95].

The same attention is required for other body districts poorly reached by the recombinant IDS enzyme administered by ERT, such as bones and the heart. As for skeletal dysfunctions, these are widely observed in Hunter patients, who mainly show dysplasia and a limited range of motion, the latter of which can be treated with physical therapy. Orthopedic surgery is rarely applied and mainly in the case of deformity of the hip joints [92]. Dental problems are also common in MPS II, as well as severe difficulties in routine dental procedures, due to the limited opening of the jaw [92]. As for the heart, valvulopathies are prevalent in Hunter patients [53], also commonly progressing under ERT, and are regularly monitored by ECG, echocardiography, or Holter examination; they often require valve replacement due to stenosis or regurgitation [96].

Inguinal and umbilical hernias are treated with surgery, although this is often a temporary solution and recurrence typically occurs [51].

In general, all surgical interventions requiring sedation or anesthesia need to be carefully programmed, since both procedures are highly risky for these patients [92].

### 6.2. Enzyme Replacement Therapy

Enzyme replacement therapy (ERT) consists of replacing the deficient or absent enzyme with a functional recombinant version through intravenous administration. Historically, the idea of proposing the substitution of enzymes as a new therapeutic approach immediately followed the identification of specific enzyme deficiencies responsible for MPSs [97,98], with the first proof of principle being provided by the famous experiment of mutual cross-correction between fibroblasts from patients affected by MPS I and MPS II in 1968 [9].

The treatment of Fabry and Gaucher patients with ceramidetrihexosidase and cerebrosidase isolated from the human placenta represented a milestone in establishing ERT [99,100]. The same protein source could not be used in MPSs, because of a low protein abundance and proteolytic degradation [101,102]; however, the cloning of genes coding for defective enzymes allowed the development of ERT for MPSs. 

To date, two different recombinant enzymes are available for MPS II: idursulfase (Elaprase, Shire HGT, recently acquired by Takeda Pharmaceutical Co., Tokyo, Japan), approved by the US Food and Drug Administration in 2006, and idursulfase beta (Green Cross Corp, Yongin, Korea), approved by the Korea Food and Drug Administration in 2012 [103]. The two enzymes show similar biochemical and physicochemical properties and a similar organ distribution and efficacy in decreasing GAG levels in preclinical studies, with idursulfase beta exhibiting higher specific enzyme activity, faster uptake by cells, and lower anti-drug antibody formation [103,104].

After 24 weeks of treatment with both enzymes, a significant reduction of uGAGs and liver and spleen volumes was observed in phase I/II clinical trials, while respiratory functions, joint mobility, and the apnea-hypopnea index showed limited or no benefits [105,106]. Some improvement in the six-minute walk test (6MWT) was observed with idursulfase-beta [105]. 

Similarly, a phase II/III clinical trial with idursulfase at the dosage of 0.5 mg/kg every week for 1 year showed a uGAG decrease of up to 52%, a reduction of the spleen and liver volume in 25% of patients, and the amelioration of 6MWT, with a limited effect on pulmonary functions, joint mobility, and the apnea-hypopnea index [107]. Furthermore, treatment with the same dosage of idursulfase beta for 1 year produced a reduction in uGAGs, without effects on developmental delay [105]. A long-term study in patients treated with idursulfase for 3 years showed an improvement in the 6MWT and in the forced vital capacity, with the latter only in treated patients younger than 18 years of age [108]. Similarly, Lampe and co-workers observed a reduction in the frequency of respiratory infections and stabilization of skeletal and cardiac disease in a study conducted in 22 neuropathic MPS II patients treated with idursulfase for 2 years [109]. However, in a follow-up study after 9 years of idursulfase therapy in 17 patients, no improvements in the respiratory function and eye, skeletal, and CNS disease were measured [110].

Concerning safety, ERT is considered to be well-tolerated, with most adverse effects being mild or moderate [111]. Although about two-thirds of patients experience an infusion-related reaction, the clinical impact of this is unclear [112]. 

A systematic evidence review on treatment of MPS II, commissioned by the American College of Medical Genetics and Genomics (ACMG) foundation, was published in 2017 [112]. The evidence review showed that ERT with weekly i.v. idursulfase infusions generally reduced uGAG levels and the liver/spleen volume in MPS II patients, while for the other outcomes, evidence was less clear. The authors also evidenced the presence of important gaps of knowledge for ERT-treated patients, including data on long-term outcomes, practical measures of progression, benefits and harms of early treatment, and patient-centered outcome (e.g., function, pain, and quality of life). Additionally, a consensus on the selection of critical outcomes and measures for evaluating treatment effectiveness and a clear definition of what constitutes “improvement” are still lacking [112].

The limited efficacy of ERT in some tissues can be explained by the low bioavailability of the therapeutic enzyme due to the low vascularization of tissues as bone, cartilage, and cardiac valves, and by the presence of biological barriers, such as the blood–brain barrier for CNS treatment [102]. Moreover, in contrast to natural continuous enzyme production, the injection of recombinant enzymes acts as a bolus and the enzyme is immediately eliminated after the infusion has finished [102,113]. This implies both a reduction in efficacy compared to a continuous administration, as demonstrated in MPS IIIA [114], and the need for frequent infusions.

The therapeutic efficacy can also be mitigated by immunoreactivity. In a previous study, more than 50% of the patients developed anti-idursulfase IgG antibodies, and 21% to 35% of these patients developed neutralizing IgG antibodies, which were associated, in some cases, with a lower reduction of uGAGs and lower improvements in pulmonary functions [108,115]. Furthermore, an association between severe mutations and the risk of developing anti-idursulfase antibodies and neutralizing antibodies has been proposed [103]. Moreover, the late initiation of treatment seems to have a great effect on reducing the therapeutic efficacy. Indeed, some pathological alterations appear very early in life or are already present during gestation [102]. If started at birth, ERT also seems to be potentially able to reduce pathology in tissue that is usually difficult to reach, such as that of the cardiac valves, bone, and brain [116]. However, a precocious diagnosis is very rare for MPS II, so therapy usually starts when pathological alterations are almost irreversible. The introduction of MPS II into newborn screening programs could be very useful for addressing this issue.

Beside efficacy-related issues, other limitations related to ERT include the need for frequent hospitalization and the high costs of therapy. The protocol presently applied plans weekly infusions, in most cases through the hospitalization of patients. This certainly reduces families’ and patients’ compliance and strongly conditions their lives, already penalized by a severe pathology, in terms of normal daily activities related to work, school, and social life, and in terms of the family budget. Fortunately, for about a decade, home therapy infusion has been available in some countries for selected patients [117,118,119], and has been shown to be a safe procedure and able to obtain a significant improvement of patients’ compliance and amelioration of their quality of life [118,120]. Therefore, the progressive inclusion of home therapy protocols should be seriously considered for the management of MPS II patients, following a period of 6 months of treatment in the hospital, to ascertain the total absence of risks [118]. 

Prices of recombinant enzymes are still very high, and are thus prohibitive for very low--income countries, where the treatment of rare disorders, such as LSDs, cannot be afforded, since not even sanitary expenses for vaccinations or primary care can be allocated.. Though the elevated costs of ERT are also becoming an important issue for high-income countries, for low- and medium-income countries, this may become a real question of whether or not to begin the therapeutic program at all [121]. 

Given the issues cited above, there has been a lot of debate on the opportunity to enroll all Hunter patients in ERT [122]. In particular, the start of ERT for severe forms of the disease is still debated, although the present position of the scientific community is that ERT must be started in all patients who do not have a more effective treatment [120]. Additionally, the discontinuation of ERT in the case of no efficacy, following an agreement with parents, has long been debated by physicians [122]. At present, in most European countries, the discontinuation of ERT is not planned, while in Canada, it can be applied in certain provinces [122]. One example of discontinuation was reported in the literature by Jurecka and co-workers [123], who, after ERT suspension for 2–8 months, observed a significant worsening of the patients’ clinical status. Therefore, as previously reported for Pompe disease [124], the dose reduction or discontinuation of ERT in MPS II patients was discouraged by the authors [123], who instead suggested the initiation of ERT, especially in severe Hunter patients, only after an accurate evaluation.

#### Improvements of ERT Traditional Protocol 

To overcome the limitations of the ERT approach, some modifications to the traditional ERT protocol have been tested, including changes of the administration route, the introduction of modified fusion proteins, and the use of alternative hosts for enzyme production.

With the aim of making ERT efficacious for the CNS compartment, different routes have been tested, including intracerebroventricular (ICV) and intrathecal (IT) administrations. ICV administration in MPS II murine models of 30 μg of idursulfase beta, every 4 weeks for 24 weeks, produced a reduction of HS in CSF and brain tissue and a significant improvement in the memory/learning functions evaluated by open-field and fear-conditioning tests [125]. A phase I/II clinical trial with idursulfase beta delivered by ICV administration is ongoing [126]. As for intrathecal (IT) administration, the positive results obtained in animal models [127] opened the way to clinical studies. A phase I/II clinical trial, consisting of the monthly administration of recombinant IDS for 6 months via an intrathecal drug delivery device (IDDD), was performed enrolling 16 children (3–18 years of age) regularly undergoing ERT, who received a dose of 1, 10, or 30 mg of idursulfase by IT or were left untreated. An 80%–90% reduction of CSF GAGs was obtained, but more than 80% of the patients showed serious adverse effects related to the IDDD. The difficulties experienced by patients required surgical revision or removal of the IDDD in 50% of cases, and mainly consisted of complications related to device insertion, device dislocation/connection issues, device breakage/malfunction/failure, implant site infection, procedural pain, and wound dehiscence. However, these severe side effects were unrelated to the use of the enzyme, which was well-tolerated (ClinicalTrials.gov identifier: NCT00920647) [128]. An extension study was conducted, enrolling 15 patients who had received monthly idursulfase-IT injections (10 or 30 mg per injection) for a median of 205 weeks. Among the five patients who had serial post-baseline general conceptual ability scores, three presented stable scores, one became untestable, and one had a worse score with respect to the baseline (ClinicalTrials.gov identifier: NCT01506141) [129]. A multicenter study, phase II/III, was completed, which enrolled 49 patients with mild or moderate cognitive impairment; they were randomized to receive 10 mg idursulfase-IT once-monthly via a surgically implanted IDDD or lumbar puncture, or no intrathecal treatment (ClinicalTrials.gov identifier: NCT02055118) [130]. The results of this study are not yet available, as well as those of the phase II/III extension study aimed at following-up patients for 148 weeks, which is expected to have results in 2022 (ClinicalTrials.gov identifier: NCT02412787) [126]. 

Attempts to cross the BBB also include the use of modified fusion proteins exploiting the concept of a molecular Trojan Horse (TH). A molecular TH is an endogenous molecule (generally a peptide or monoclonal antibody) which, by binding an endogenous receptor located on the luminal side of brain capillary endothelial cells, can be transported across the BBB through receptor-mediated transport. The conjugation of a therapeutic recombinant protein to a TH enables the transport of the protein drug across the BBB. The receptors mainly used for this purpose are insulin receptors (IR) and transferrin receptors (TfR), which transport circulating insulin and transferrin across the BBB, respectively, in addition to receptor-specific monoclonal antibodies [131,132]. The insulin receptor was exploited as a fusion protein with IDS in Rhesus monkeys, showing a safety profile for chronic treatment with weekly intravenous infusions of 3–30 mg/kg of drug [133]. As for the TfR, treating mice with the human IDS enzyme fused with the monoclonal antibody against the mouse TfR allowed an uptake of the fusion protein by the brain and spinal cord 100-fold greater than the uptake of IDS alone [134,135], together with an amelioration of brain GAG deposits and the maintenance of cognitive functions. The pharmacokinetics, safety, and potential efficacy of this strategy were evaluated in a first-in-human study in 14 patients with MPS II [136]. In a dose-escalation study performed in two patients, drug concentrations in the plasma were dose-dependent and peaked at 3 h post-infusion, and no or only mild adverse reactions were exhibited. Both plasma and urine levels of HS and DS were reduced, as well as HS levels in cerebrospinal fluid. Two patients showed some amelioration of the neurocognitive and motor symptoms. 

Finally, with the aim of reducing the costs of ERT or producing proteins with a potentially improved stability and pharmacokinetic and pharmacodynamic properties, alternative hosts have been evaluated for the production of recombinant enzymes [137]. The yeast *Pichia pastoris* and the bacterium *Escherichia coli* K12 have been used for the expression of IDS [138,139,140,141,142]. Although active, these enzymes need to undergo preclinical evaluations before being proposed for ERT.

### 6.3. Haematopoietic Stem Cell Transplantation (HSCT)

HSCT consists of the transplantation of blood stem cells from a compatible healthy donor to a patient, with bone marrow peripheral blood or umbilical cord blood usually being used as the source of blood stem cells. The transplanted cells and/or their progeny can become local permanent sources of functional therapeutic enzymes. Since monocytic/phagocytic cells are able to cross the BBB and host in the brain as microglia, microglia derived from donor cells would be able to secrete the deficient enzyme that would be captured by receptor neurons correcting enzyme deficiency. Araya et al. [143] demonstrated the presence of donor cells in the microglia of patients with MPS II 10 months after transplantation, not only in the perivascular spaces, but also in the cerebral parenchyma, suggesting the potential of HSCT for treating the neurological symptoms of Hunter syndrome. Moreover, if full donor chimerism is achieved, a single intervention could provide a durable lifelong enzyme source to the affected patient [144]. The consequent significant reduction of hospitalization would importantly ameliorate patients’ and families’ compliance. Finally, with its one-time administration, HSCT would significantly help to reduce costs of treatment with respect to ERT weekly administrations. Notwithstanding these great advantages, many safety issues, related to the high risk/benefit ratio of the transplant procedure and immunosuppressive regimens, and the risk of graft vs. host disease (GvHD), due to the need to use heterologous donors, need to be considered. 

HSCT was applied for the first time for MPS II in 1986 to a 7 year old patient [145]; even if the intellectual ability was stabilized after transplantation, the plasma enzyme levels remained far below normal and 3.5 years after transplantation, the patient died due to cardiovascular complications [146]. Since then, and in particular after the introduction of ERT, HSCT has been discouraged and rarely performed in most Western countries. Therefore, the clinical experience with HSCT in MPS II is small, especially compared to MPS I, for which more than 600 transplantations have been performed. Available data on HSCT in MPS II are outdated or reveal poor patient selection criteria (especially concerning the presence of neurological symptoms and the age at transplantation) and a great variability of cell sources, conditioning regimens, and outcomes of interest [146]. A study performed by Tanaka and colleagues, evaluating the long effects of HSCT, showed an improvement in uGAG levels, heart valve regurgitation, brain magnetic resonance imaging (MRI) atrophy, category I and II brain lesions, and activity of daily living (ADL). However, these ameliorations were only observed in patients treated before the development of brain atrophy and cardiac valve regurgitation [147]. After HSCT, an improvement in motor and speech skills was reported in patients with a severe MPS II phenotype, although an incidence of graft versus host disease of 41.1% was observed [148]. A patient who underwent umbilical cord donor HSCT at 70 days of age was reported to have a normal growth chart and an improvement in cognitive and communication skills, evaluated 7 years post-transplant [149]. Moreover, the HSCT impact on height and weight was found to be the same as that of ERT [150]. A retrospective study reported the effects of HSCT in 146 MPS II patients compared with 51 patients treated with ERT only and 15 untreated patients. DS and HS levels were reduced in all treated patients; for severe patients, the ERT/HSCT combined therapy produced a greater reduction of DS and diHS-0S compared with ERT treatment. HSCT patients showed stable or improved brain lesions after transplantation. Graft versus host disease occurred in eight (9%) out of 85 cases, and nine (8%) patients died from transplantation-associated complications [151].

Although the application of HSCT to MPS II still remains controversial, the protocol refinement and risk reduction progressively being obtained for HSCT are leading professionals to reconsider the application of transplants for the treatment of Hunter patients, which is now a therapeutic option offered in some countries, including Japan [151], China [148], and Brazil [152], while it is not routinely used in the United States [112]. More recently, programs of ex vivo HSCT gene therapy allowing the use of autologous cells, thus reducing the patients’ need for medication, and increasing the transfer efficiency, have been tested in several LSD animal models [144] and will be detailed in the next paragraph. However, it has been hypothesized that the long timing required for the transplanted cells to cross the blood–brain barrier and to obtain progressive cell replacement, of about 1 year, may somehow impede the treatment efficacy for a long time post-transplant, letting overall brain disease progression prevail over the benefits of the treatment [144]. All this considered, it becomes evident that amelioration of the transplant procedure, reducing its risks of morbidity/mortality, should be accompanied by a very early detection of Hunter patients in the population, which could be obtained through inclusion of the disease in the expanded screening programs.

### 6.4. Gene Therapy

MPS II, as well as other MPSs and most LSDs, have several features that make them potential candidates for gene therapy. They are monogenic with a well-known pathophysiology, at least from a biochemical point of view, and can benefit from systemic cross-correction. Through this mechanism, the enzyme eventually produced and released by the cells of a depot organ, after gene modification, can be taken up by other cells and organs. Moreover, the levels of induced gene expression are generally not critical enough to have a positive impact on clinical manifestations, with 5%–15% of the normal enzyme level being required to maintain a healthy condition [111].

Gene therapy approaches can be divided into two different strategies: in vivo gene therapy and ex vivo gene therapy. In vivo gene therapy consists of the direct infusion of the vector carrying the therapeutic gene into the patient’s body; in ex vivo gene therapy, cells derived from the recipient patient, usually hematopoietic stem cells or peripheral blood cells, are extracted, transduced in vitro with the therapeutic gene, and infused back to the patient. In addition, gene therapy approaches can be classified according to the type of vector used (viral or non-viral) and according to the administration route used, including intravenous, intramuscular, intracerebral, or intra-CSF (lateral ventricle, cisterna magna, or intrathecal lumbar injection) administration. For in vivo gene therapy, the genes are mainly transduced by using different viral vector systems, including retroviral, lentiviral, adenoviral, and adeno-associated virus (AAV)-based vectors, as well as non-viral vectors. For ex vivo gene therapy, retrovirus and lentiviral vectors are mainly used [153].

Gene therapy has been considered a therapeutic option for several LSDs [154]; the different strategies used for MPS II, together with their main features, are described in the following paragraphs.

#### 6.4.1. Retroviral Vectors

Retroviral vectors can integrate into host genomes in dividing targeted cells, such as hematopoietic cells. Therefore, the therapeutic gene can be transmitted and expressed for a long time in daughter cells. However, the integration can potentially cause mutagenesis and tumorigenesis in targeted cells [155]. They were the first vectors used for MPS II. Although some positive results were obtained in vitro [156,157], also leading to a clinical trial (ClinicalTrials.gov identifier: NCT00004454), this approach failed due to short-term gene expression and adverse effects [111]. 

#### 6.4.2. Adeno-Associated Viral Vectors

Adeno-associated viruses are small, replication-defective viruses, requiring a so-called helper virus, such as an adenovirus or herpesvirus, to replicate. Since they do not integrate into the host genome and persist as episomes, there is a low risk of insertional mutagenesis and genotoxicity [158], even if some exceptions exist [159,160]. AAV vectors can transduce a wide range of non-dividing and dividing cells, providing long-term transgene expression in non-dividing cells. The main limitations are represented by delayed expression in transduced cells and the small packaging capacity [161,162,163].

Thanks to their stability, long-term expression, and low immunogenicity, AAVs are the most used viral tool in gene therapy for MPSs. The first attempts at using gene therapy based on AAV vectors in MPS II consisted of the use of AAV2/8 vectors, which were administered intravenously to adult MPS II mice. A complete rescue of the enzymatic activity and a full clearance of GAG storage in the plasma, spleen, lung, heart, kidney, brain, and muscle, as well as a normalization of skeletal malformations, were observed [164,165]. However, as observed in other MPSs, the AAV9 was later shown to be the most promising serotype. An AAV9 viral vector carrying the *IDS* gene has been administered to CSF through intracisternal injections in MPS II mice producing, 4 months after treatment, a significant increase in IDS activity throughout the encephalon with a reversal of CNS pathology [166]. The same results were obtained by two subsequent studies [167,168], using a similar strategy, but via intracerebroventricular injections. Laoharawee et al. observed supraphysiological levels of IDS in the circulation (160-fold higher than wild-type) for at least 28 weeks post-injection and in most peripheral organs (up to 270-fold) at 10 months post-injection, but only low levels (7% to 40% of wild-type) in all areas of the brain. Nevertheless, this was enough to prevent brain pathology and neurocognitive impairment [168]. 

These positive preclinical results opened the way to the development of a clinical trial for the treatment of CNS manifestations in MPS II, based on the AAV9 vector RGX-121 produced by Regenxbio Inc. and administered by intracisternal injections. It is a phase I/II multicenter, open-label, dose-escalation study, still in the recruiting phase (ClinicalTrials.gov identifier: NCT03566043). 

#### 6.4.3. Lentiviral Vector

Lentiviral vectors integrate into the host genome and provide stable gene expression. Unlike retrovirus vectors, they can mediate gene transfer into dividing cells, as hematopoietic stem cells, but also non-dividing cells, as nerve cells [169]. They are mostly used for ex vivo gene therapy, for which they enable a significantly improved HSC transduction efficiency and therapeutic gene expression [170].

In the MPS II mouse model, lentiviral ex vivo hematopoietic stem cell gene therapy produced a slight, significant increase of IDS activity in cerebral tissues (2.9% with respect to wild-type controls), 24 weeks post-treatment, and ameliorated primary and secondary lysosomal storage and autophagic dysfunction in the brain and visceral organs. Furthermore, the treatment prevented the deterioration of neuronal functions observed in untreated MPS II mice. [171].

#### 6.4.4. Non-Viral Gene Therapy

Non-viral vectors may overcome the toxicity problems associated with viral vectors, although they suffer from a low gene transfer efficiency and low gene expression levels. To improve the gene expression level, DNA can be transferred to the tissue ‘naked’ or combined with several physical and chemical methods [21]. 

The only non-viral gene therapy approach tested in MPS II has been electro gene transfer (EGT), applied for gene delivery in the skeletal muscle of MPS II mice. Although an elevated production of the protein was obtained inside the muscle, a limited release in plasma and a strong anti-IDS immune response, with a limited therapeutic effect, were observed [172].

#### 6.4.5. Genome Editing

Genome editing is an innovative genetic engineering tool in which DNA is inserted, deleted, modified, or replaced in the genome of a living organism. In this strategy, a site-specific engineered endonuclease (such as zinc finger nuclease (ZFN), transcription activator-like effector-based nuclease (TALEN), and CRISPR/Cas9 technology) generates double-strand breaks at an appropriate position in the genome, which are repaired through either non homologous end joining or homologous recombination. As a therapeutic tool, this system is commonly used in combination with delivery vectors engineered with the therapeutic gene to target disease tissue [173].

As for MPS II, the *IDS* gene was inserted at the albumin locus using AAV8 vector-mediated delivery and ZFN-mediated site-specific insertion, providing a robust IDS expression in a wild-type mouse [174]. In MPS II mice, the administration of an AAV8 vector with albumin locus-targeting ZFN in hepatocytes induced dose-dependent elevation of the IDS enzyme in blood and other peripheral tissues. This treatment reduced GAG storage, and DS and HS levels in visceral organs and the brain. The treatment also prevented the neurocognitive deficits observed in untreated mice [168]. These positive results led to the development of a phase I/II clinical trial, with the first patient being treated with in vivo gene editing therapy (ClinicalTrials.gov identifier: NCT03041324) [175]. The trial included three cohorts with different dosages. Sixteen weeks post-gene therapy, the medium-dose group showed urinary HS and DS reduction from the baseline; however, plasma IDS enzyme activity was not detected by the fluorometric assay [176].

### 6.5. Cellular Therapy and Nanocarriers

One of the first treatments evaluated for MPS II was plasma infusion [177]. The good clinical and biochemical results obtained in the study, together with the cross-correction demonstrated by Fratantoni [9], led Knudson and colleagues to carry out the first cellular therapy study for MPS II, based on leukocyte transfusion from an unaffected donor [178]. They reported a good long-term improvement of the transfused patient, with both a transient decrease of GAG storage and a reduction of different clinical parameters, like joint mobility and abdominal size. Based on these results, since 1975, the transplantation of healthy fibroblasts has also been tested [179,180,181]. It has allowed an increased IDS activity to be detected, but unfortunately without any clinical benefits. Amnion membrane implantation was evaluated, but without success [182]. In 2005, thanks to the availability of the mouse model, another cell therapy study was conducted [183]. Friso and colleagues reported positive results of the intraperitoneal implantation of alginate microcapsules carrying C2C12 murine myoblasts over-expressing IDS. They demonstrated increased enzyme activity in the plasma and organs of treated mice, as well as a reduction of GAGs in urine and tissues [183]. 

After this, no other cell therapy studies were reported for MPS II, as opposed to the use of carriers and nanocarriers. Brain-targeted PLGA-nanoparticles were reported to be able to carry a high molecular weight model drug (albumin) across the blood–brain barrier in MPS I and MPS II mouse models [184]. Recently, the same nanoparticles were demonstrated to be able to transport the human recombinant IDS enzyme into the central nervous system, leading to a reduction of GAG storage and neuroinflammation in the MPS II mouse model [185]. 

### 6.6. Substrate Reduction Therapy

Substrate reduction therapy (SRT) aims to prevent storage not by correcting the original enzymatic defect, but by reducing the synthesis of the accumulate substrates. Usually, SRT drugs are analogues of synthesis intermediates acting as inhibitors of the anabolic enzymes [186]. 

Currently, SRT has been approved to treat some LSD, including Gaucher disease type I and Niemann Pick type C disease. As for MPSs, it is difficult to find nontoxic inhibitors for the enzymes involved in GAG synthesis; indeed, intermediates are carbohydrates or their derivatives which are involved in many other metabolic pathways and analogues of these compounds would probably interfere with other cellular processes. Therefore, currently developing strategies are based on the indirect inhibition of GAG synthesis [111].

In 2006, genistein (4′,5,7-Trihydroxyisoflavone), a natural isoflavone purified from soya beans, was identified as able to induce a reduction of GAG storage in fibroblasts from MPS I, MPS II, MPS IIIA, and MPS IIIB [187]. In 2010, in the MPS II mouse model, a 10-week treatment with genistein at 5 or 25 mg/kg/day produced a reduction of GAG levels in the urine, liver, spleen, kidney, heart, and brain for some animals [188]. In MPS II patients, an improvement in the connective tissue elasticity and joint range of motion was observed after 26 weeks of treatment with 5 mg/kg/day of genistein [189]. Originally, these effects were thought to be mediated by the inhibition of the epithelial growth factor (EGF) receptor, with EGF being required for GAG synthesis [190]. More recently, genistein was also reported to partially correct the cell cycle alterations observed in MPS II cells [191]. 

Considering the ability of genistein to cross the BBB, this SRT compound could potentially be used for a combined therapeutic approach with ERT. However, the clinical trials performed on MPS III subjects have not given encouraging results for a possible therapeutic effect of genistein on CNS symptoms [111].

### 6.7. Pharmacological Chaperone Therapy

Some mutations associated with lysosomal enzymes can produce misfolded proteins, leading to their retention in the endoplasmic reticulum (ER) or Golgi apparatus, defective transport to lysosomes, and degradation by the ubiquitin-proteasome pathway (known as ER-associated degradation (ERAD)).

Pharmacological chaperone therapy (PCT) is based on the use of small-molecule drugs able to interact with misfolded mutated enzymes, favoring their correct folding and intracellular trafficking, and enhancing their stability and enzymatic activity [192]. These molecules are usually substrate analogues which work at low concentrations (lower than 10 µM), can be orally administrated, are not immunogenic, and have been shown to act in the brain in preclinical and clinical studies [111,193,194,195]. On the other hand, they can only be used for a limited number of mutations and can produce off-target adverse effects and enzyme inhibition [111].

LSDs can be considered good candidates for PCT since minimal increases in activity are considered to be sufficient for positively impacting the phenotype [192]. Indeed, PCT has been considered for the treatment of several LSDs [192], including MPSs [196], and has been approved for clinical use for Fabry disease [197]. Since, for IDS protein, some mutations have been reported, causing misfolding, ER retention, and degradation by ERAD [198], PCT has recently also been investigated for MPS II. The Δ-unsaturated 2-sulfouronic acid-N-sulfoglucosamine (D2S0), a sulphated disaccharide derived from heparin, was reported to increase the thermal stability of human IDS in vitro. It was also able to increase the residual activity of mutant IDS in patient fibroblasts and mutant IDS derived from six different mutations in a transient gene expression system [199]. 

Although promising, the studies on this therapeutic strategy for MPS II are still in a very preliminary phase.

## 7. MPS II Pathogenesis: In Vitro Evaluations and Animal Models

Following the discovery of MPS II genetic and biochemical bases, most research efforts were addressed to identify possible therapeutic strategies and to reach adequate clinical management of the patients. However, many studies, involving in vitro and in vivo models, have been performed with the aim of identifying the cellular and physiological processes which, starting from the genetic defect, are responsible for the observed pathological alterations. Although, for many years, MPS II pathogenesis was only considered the result of undegraded GAG accumulation in different organs, it is now showing other aspects, previously unsuspected, which might explain the overall complexity of the disease. In recent years, evidence has emerged which indicates that the signs and symptoms of Hunter disease are not only due to a general engorgement of the cells and extracellular matrix, progressively leading to cell death and general organ impairment, but also to the alteration of different signaling pathways, including fibroblast growth factor [200] or sonic hedgehog [201], independently of the primary storage. 

This section describes the studies involving in vitro and in vivo models performed with the aim of dissecting MPS II pathogenesis.

### 7.1. Cell Models

The main studies on MPS II started in the ‘60s and at that time, available models were almost only cells. When Hunter syndrome was still considered the X-linked form of MPS I, Danes and Bearn demonstrated that fibroblasts from patients’ biopsy well-recapitulated GAG storage [202,203], opening the way for their use as a disease model and allowing the discovery of the biochemical basis of the disease, through previously cited studies [7]. From then on, fibroblasts have become a basic tool for studying the disease, from a diagnostic and therapeutic point of view, and for understanding the disease itself. Since the late ‘90s, fibroblasts have also been used to test the therapeutic activity of IDS enzymes released and purified from IDS-overexpressing cells [204,205], as well as in all the in vitro tests conducted as a preliminary analysis in the development of new therapeutic options, for instance, genistein [188,206], new recombinant human IDS enzymes [103], anti-human transferrin receptor antibody IDS fusion proteins [135], and brain-targeted nanoparticles loaded with IDS enzymes [185].

The use of fibroblasts from MPS II patients and healthy controls allowed basic affected mechanisms to be dissected. Mazzoccoli and colleagues demonstrated the altered expression of clock genes and clock-controlled genes, suggesting their possible involvement in the deregulation of cellular homeostasis and therefore in the pathophysiology of the disease [207]. Recently, Moskot and co-workers demonstrated a cell cycle block of MPS II cells in the G0/G1 phase, compared to healthy controls, and a partial capability of genistein to rescue these cell cycle disturbances [191]. 

In the last decade, given the inability of recombinant enzymes to cross the BBB and the consequent need to better understand the brain disease pathophysiology, with the aim of possibly identifying new potential therapeutic targets, there has been a growing interest in discerning the neurological pathology of Hunter syndrome. Fusar Poli and colleagues used neural stem cells (NSCs) from the MPS II mouse model to study the mechanism leading to the neuronal defect. They highlighted an earlier differentiation of MPS II NSCs into different neurological cell types, compared to controls, with a huge lysosomal aggregation in all glial cells, which also displayed an increased apoptosis [208]. The same research team later evidenced that neuroinflammation precedes glial degeneration, together with the appearance of oxidative damage and the impairment of mitochondria function, finally leading to neuronal apoptosis and death [209]. Treating MPS II astrocytes with alpha-tocopherol or low oxygen conditions may revert this pathological phenotype, thus paving the way to new possible treatments, complementing ERT in addressing the oxidative stress [209]. 

In recent years, induced pluripotent stem cell (iPSC) technology, allowing adult cells to be re-programmed to immature cells and then differentiated into potentially all cell types, has been exploited to study MPS II pathophysiology. Varga and co-workers published the successful obtainment of four different iPSC lines starting from peripheral blood mononuclear cells (PBMCs) of three Hunter male patients (1, 3, and 7 years old) and one MPS II unaffected female carrier [210,211,212,213], while Hong generated iPSCs starting from fibroblasts of a 3-year-old patient with a frameshift mutation in the *IDS* gene [214]. Very recently, Rybová demonstrated the ability of MPS II iPSCs to differentiate to neuronal lineages, reflecting the human and mouse phenotype with reduced IDS activity, increased GAG storage, and increased lysosomal membrane marker LAMP1 and lysosomal luminal marker CatD [215]. Finally, in 2019, Kobolak and co-workers differentiated the four iPSCs lines obtained by Vargas and described above, into four lines of neuronal progenitor cells, which were then terminally differentiated into cortical neurons. Both cell types were then evaluated for their neuronal features, and their potential use as an in vitro cell model of the neuronal phenotype of MPS II disease was assessed [216]. MPS II iPSCs will be very useful for future studies since they allow tissue-specific cells to be obtained, and therefore permit not only the study of the pathophysiology of the disease, but also the performance of in vitro studies for the analysis of new potentially therapeutic molecules using suitable types of cells, which are always patient-derived. Finally, iPSCs could be used to create a human BBB model for in vitro “brain-targeting” studies, allowing more reliable results.

### 7.2. Animal Models 

#### 7.2.1. Mouse Model

In the last 30 years, the use of mouse models for biomedical research has seen an escalation, and was further accelerated by the sequencing of the murine genome in 2002 [217].

**Figure 3:**
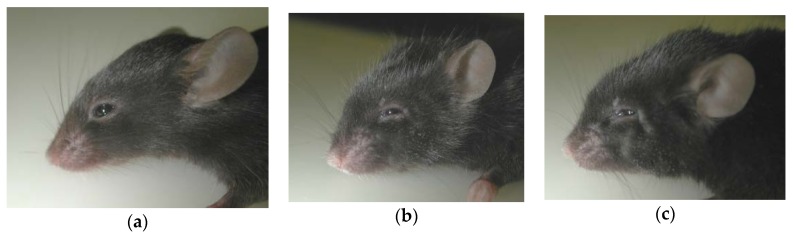
*Ids-*ko mice of 9 (**b**) and 14 (**c**) months of age, derived from the first mucopolysaccharidosis (MPS) II murine model [19,218], compared to a wild-type mouse of 9 months of age (**a**). *Ids*-ko mice show the progressive worsening of coarse fur, as well as distorted facies and broadened snouts, as a result of sclerosis and enlargement of the bones of the skull [219].

Concerning Hunter syndrome, Muenzer described the first knock-out (ko) mouse for the *Ids* gene in a congress report in 1999 [19] (Table 2). This *Ids*-ko mouse was created by replacing part of exon 4 and the entire exon 5 of the murine *Ids* gene with the neomycin resistance gene. In 2002, the first characterization of the model was published, demonstrating that it well reflects the human pathology, with a loss of the enzyme activity, elevated GAGs in the urine and some organs (liver, kidney, and heart), and skeletal deformities and vacuolization in many tissues [218] (Figure 3). In the same work, the authors also reported the first use of the *Ids*-ko mouse model for the pre-clinical trial of ERT. From there on, this model was further characterized by different groups. In the subsequent years, two independent groups confirmed the data published by Muenzer and the reliability of this model [164,183]. Moreover, Cardone and co-workers amplified the characterization of the model, analysing and confirming the presence of elevated GAG storage in other organs (spleen, lung, muscle, and brain). They also highlighted widespread vacuolization in the *Ids*-ko brain, as well as an elevated number of cells positive to the lysosomal marker Lamp-2. Even the walking pattern and the behavior in the open-field test were impaired [164]. In 2007, Muenzer and colleagues published an extensive evaluation of their model, reporting an increased weight of almost all organs at 40 weeks of age, elevated GAGs in urine and organs starting from 4-7 weeks of age and throughout the lifespan, the spread and progression of skeletal deformities with a decline in activity, and a reduced lifespan of about 1 year. Histologically, they saw cell vacuolization starting at 4 weeks, neuronal necrosis in the brainstem and spinal cord, an elevated GAG content, and Lamp-1-positive cells [219].

In the last decade, different groups have carried out studies to better understand the MPS II pathology, especially the neurological one, and to find new markers and parameters to monitor during pre-clinical trials. They have reported a disorganized neuronal pattern in the brain, an increase in glial apoptotic cells preceding neuronal death together with a reduced number of PDGFR-α-positive glial progenitors [208], and an acute inflammatory state in 8-day-old mice, and progressive astrogliosis and microgliosis (up to 8 months) [209]. Additionally, 8-month-old mice showed extensive neuroinflammation, demonstrated by elevated cytokines levels [223]. An on-tissue high spatial resolution MALDI IMS analysis, followed by GM3-immunohistochemistry, was able to detect GM2 and GM3 ganglioside accumulation throughout 16 brain regions, with a distribution similar to GAG Alcian blue staining [224]. Skeletal, motor, and cognitive defects were also confirmed by X-ray imaging and a time-course behavioral analysis at 2, 4, 6, and 8 months, when exposing mice to the subsequent tests: open-field test, spontaneous alternation, inverted screen test, horizontal bar test, elevated plus maze test, sociability and social novelty preference, and rotarod motor learning. Despite the X-ray imaging confirming progressive skeletal impairment starting as early as 2 months, the behavioral analysis only highlighted a reduced activity and performance in the 8-month-old mice [225]. The behavioral impairment was also confirmed by another study using the open-field and inhibitory avoidance tests, but evidencing defects starting as early as 6 months [226]. 

To better understand the neurological pathology, Salvalaio and colleagues carried out an RNA-seq analysis in the cerebral cortex and midbrain/diencephalon/hippocampus areas of *Ids*-ko mice and wild-type controls at 9 months of age. They highlighted several pathways already reported as impaired in other LSDs or neurodegenerative disorders, but never in the MPS II mouse, including calcium signaling, synapse and neuroactive ligand-receptor interaction, axon guidance, circadian rhythm, Wnt signaling, autophagy, and the immune and inflammatory system. A dysregulation of oxidative stress and an involvement of mitochondria were also confirmed [227].

In the last 10 years, another three mouse models have been developed, characterized, and used for pathophysiology and treatment studies (Table 2). In 2010, Jung and colleagues reported a new *Ids*-ko mouse created by replacing 1485 bp of exon 2 and exon 3 of the *Ids* gene with the neomycin resistance gene [165]. They characterized this model, confirming most of the features already reported by Muenzer, but a little bit earlier in life, suggesting that this may be due to the location of the deletion. They reported glycogen depletion in mouse hepatocytes, inversely proportional to GAG storage and reversible by ERT [228]. They highlighted hearing loss and the presence of exudates in the middle ear starting at 17 weeks, which were treatable with ERT, even if unable to revert the damage of the microstructure of the inner ear [229]. Very recently, they further characterized the brain pathology, demonstrating the presence of progressive autophagy starting at 3 months, characterized by an increased number of p62 and SCMAS-positive cells in different brain regions (cerebral cortex, cerebellum, hippocampus, thalamus, and amygdala) and in three cellular types (neurons, microglia, and pericytes) [230].

In 2012 and 2016, two further MPS II mouse models were reported, having the same deletion of the *Ids* gene from exon 2 to exon 5, respectively produced by JCR Pharmaceuticals Co., Ltd. (Hyogo, Japan) [222] and Taconic Biosciences (#TF1838) [166]. Both models share the same main phenotypic characteristics as previously reported *Ids*-ko models, thus making all four *Ids*-ko mice good disease models for both pathogenesis and treatment-efficacy preclinical studies.

In the last twenty years, all these *Ids*-ko mouse models have been widely used for preclinical studies on different types of treatments. Among these are different dosages and routes of administration of ERT [103,125,222,229,231,232,233,234,235], microcapsules enclosing myoblasts over-expressing IDS [183], different gene-therapy approaches [164,165,166,171,172,223,236], genistein [188], brain-penetrating IgG-Iduronate 2-sulphatase fusion protein [134], HSCT [234,237,238], ZFN-mediated in vivo genome editing [239], engineered nanoparticles for IDS enzyme brain-targeting [184,185], anti-human transferrin receptor antibody fusion protein [135], and chloroquine [230]. They were also useful for testing new methods of GAG analysis, aimed at improving their use as biomarkers in pre-clinical studies and successively in patients [125,223,240,241]. In particular, the RP-HPLC method [242] was applied to analyse the total relative amount of HS and its disaccharide composition [223], while another HPLC-based approach, founded on an analysis of the 2-sulfoiduronic acid derived from the non-reducing end of GAGs, allowed clear discrimination between *Ids*-ko and wild-type mice in the liver and brain [240]. Recently, two different studies reported the use of mass spectrometry for GAG analysis in MPS II mice. The first one highlighted the appropriateness of using HS, measured by LC-MS/MS, as a biomarker in the brain and CSF, which is more sensible compared to the total amount of GAGs measured by standard techniques; they demonstrated a positive correlation of HS content in CSF with HS and total GAG levels in the brains, which is very useful from a clinical perspective [125]. Finally, Menkovic and co-workers reported a UPLC–MS/MS approach for the absolute quantification of HS and DS in many tissues, with an increase of both GAGs in all of them, particularly HS [241].

#### 7.2.2. Dog Spontaneous Model

The first animal model for MPS II was described in 1998 (Table 2) [220]. It was a Labrador Retriever dog model, showing progressive incoordination, hepatomegaly, osteopenia, multifocal corneal opacity, asymmetric ataxia, labial thickening, and elevated uGAGs. MPS II diagnosis was confirmed biochemically and supported by the mother’s carrier status. However, this spontaneous mutant did not generate offspring and the analysed littermates were all unaffected, so it remained an isolated case.

#### 7.2.3. Zebrafish Model

In 2010, Moro and colleagues reported the first model for Hunter syndrome in *Danio rerio* (zebrafish) (Table 2) [221]. In this work, they isolated and characterized, for the first time, the *IDS* ortholog in zebrafish and demonstrated its importance in early vertebrate development by its knocking-down with antisense morpholino oligos. Half of the morphants died within 24 hours, and those who survived had several pleiotropic defects, among which were deformities of the body, head, and trunk. *IDS* morphants showed reduced IDS activity leading to an impaired dorso-ventral gene expression, with the expansion of the mesendodermal portion in embryos (increase of *eve1* and *sox17*), an increase of the TGFβ signaling, and craniofacial defects and impaired facial cartilage development with reduced *sox10* and *crestin* marker expression at 5 dpf (days post-fertilization) [221]. The morphants also showed aberrant heart development and atrioventricular valve formation, due to disrupted Shh signaling in early life stages, as well as an increase of the Wnt/β-catenin pathway, which were both due to IDS loss of function. These data were confirmed in mice, where GAG storage is limited in the heart at postnatal stages. This demonstrated a central role of these two pathways in the cardiac pathogenesis of Hunter syndrome [243]. Recently, the same group reported an analysis of bone development and demonstrated an involvement of the FGF pathway in early stages, which also anticipates the accumulation of detectable GAGs [200]. They first described dysregulation of FGF signaling in 2 and 6 dpf *IDS* morphants. In this work, they subsequently developed a stable knock-out zebrafish model for MPS II by using the CRISPR/Cas9 approach, creating a 5 bp deletion in the *IDS* exon 2. They confirmed, in this model, that early in life, a down-regulation of the FGF pathway and the misexpression of FGF target genes, precede skeletal deformities and GAG storage. These data were then confirmed in 1- and 2-week-old *Ids*-ko mouse models [200]. These results support the hypothesis that the *IDS* gene is involved in developmental processes, independently of GAG storage. 

## 8. Disease Biomarkers

A ‘disease biomarker’ is a measurable analyte or clinical feature used to identify a specific disease (or group of diseases) and quantify the disease burden before, during, and after treatment. 

As for MPSs, ideal biochemical biomarkers would be specific to a particular type or group of MPSs, help to discriminate more severe from less severe phenotypes, correlate with neurological involvement, respond to therapy, and be easily quantified [244]. In MPSs, primary biochemical biomarkers are represented by the primary storage material; for MPS II, they are partially degraded HS and DS fragments that accumulate in the lysosomes and extracellular matrices (ECM) and are secreted into the bloodstream and then excreted in the urine [245]. The uGAG level measured by different dye binding assays (dimethyl-methylene blue and alcian blue) is the most common biomarker used for the MPSs because of its simplicity and rapidity [246]. These methods have been extensively used in clinical trials of ERT, where they have evidenced a rapid and significant decrease of uGAGs in response to treatment [111]. However, the lack of significant changes during the long-term monitoring of treatment limits the reliability of uGAGs as disease biomarkers for MPS II, as well for other MPSs. Moreover, the dye-binding methods are aspecific and cannot discriminate between different types of GAGs. Finally, it is still being debated whether the uGAG level can be a measure of the total body burden of disease or if it only reflects the renal involvement [245].

The urinary DS to CS ratio (DS/CS) has been shown to be a more reliable candidate marker and it demonstrates some advantages if compared with total uGAGs as it does not depend on the age or hydration of the patients. Hence, DS/CS ratios quantified by 2D chromatography, followed by the semi-quantitation of extracted uGAGs, have been shown to positively respond to ERT and HSCT in MPS I, II, and VI patients [247].

In the last years, several approaches using mass spectrometry have been implemented with the aim of evaluating glycan-based molecules, as well as other types of molecules, as potential candidate biochemical biomarkers for MPS II. Among them, ESI-MS/MS was used to quantify the level of naturally occurring N-acetylhexosamine-containing mono- and disaccharides in the urine and plasma of MPS patients [248]. One of the most used methods is liquid chromatography combined with MS/MS (LC-MS/MS), with the previous depolymerization of GAG chains [249]. This approach has been used by different groups to discriminate disaccharides from HS and DS and their different sulphated forms in urine and blood. Khan evidenced increased DS and HS levels in the blood of untreated MPS II patients with respect to age-matched controls by LC-MS/MS, and showed that ERT reduced both DS and HS levels [250]. The following year, Fujitsuka and coworkers, using the same method, showed that MPS II patients treated with HSCT had lower blood levels of HS and DS than patients with ERT. Moreover, they evidenced a significant increase in patients’ blood of different pro-inflammatory factors, with some of them normalizing after HSCT [251].

The LC-MS/MS approach was also used to quantify DS or HS in cerebrospinal fluid (CSF), as a tool for monitoring neurological disease progression [252] or evaluating the efficacy of intracerebroventricular ERT [125]. Similarly, glycan reductive isotope labelling–liquid chromatography/mass spectrometry (GRIL–LC/MS) was implemented to analyze the mono-, di-, or tri-saccharide composition released from the non-reducing ends of GAG chains, after depolymerization, in different MPSs [253].

During the last years, the pathophysiology of MPS II, as well as of other LSDs, has been revealed to be more complex than expected, with intracellular and extracellular GAG accumulation likely activating secondary pathogenic pathways that may perturb the cellular and tissue homeostasis [254]. All this has encouraged the search for ‘secondary biomarkers’, i.e., analytes or features reflecting altered cellular and tissue homeostasis instead of the primary enzymatic defect [245].

Randall and colleagues demonstrated that the concentration of heparin cofactor II-thrombin complex (HCII-T) in serum was highly elevated in MPS I, II, and VI patients with respect to controls and they evidenced that HCII-T may reflect the disease severity, as well as the response to treatment [255]. Later, two different longitudinal studies confirmed serum HCII-T as a reliable disease biomarker that rapidly responds to changes in patients’ clinical status [256,257]. In addition, Clarke evidenced a correlation between serum HCII-T levels and antibodies to idursulfase in ERT-treated MPS II patients [257]. Langford-Smith evidenced that serum HCII-T responds rapidly to treatment (HSCT and ERT) in MPS I, II, and VI patients, while the urinary DS/CS ratio responds more slowly, potentially representing short-term and long-term treatment outcomes, respectively [256]. 

Recently, proteomic approaches have also been used. Heywood analyzed urine samples from GAG-positive MPS I, MPS II, and MPS VI patients by label-free proteomics, followed by a targeted proteomic multiple reaction monitoring LC-MS/MS assay; the study evidenced several differentially expressed proteins implicated in extracellular matrix organization, some of which allowed the researchers to differentiate between the MPS II neurological and non-neurological phenotype [244]. Later, the same group measured, by using the LC-MS/MS method, the levels of different forms of urinary hydroxylysine, as indicators of altered collagen metabolism and hence potential biomarkers of MPS disease [258]. A different proteomic approach based on two-dimensional gel electrophoresis combined with MALDI-TOF/TOF was used to identify the differential protein profile in the urine of MPS II patients [259].

As for clinical biomarkers, only indicators somehow reflecting the extent of the disease burden and thus its progression could be considered candidate clinical biomarkers. The following clinical measurements have been used to evaluate the efficacy of ERT in MPS II: distance of a 6-minute walking test (6MWT, as a measure of the physical functional capacity), forced vital capacity (FVC, as a measure of respiratory function), liver and spleen volumes measured by abdominal MRI, and passive joint range of motion [106,107]. Other clinical parameters are used to determine the efficacy of the neurological compartment and generally, they are based on scales that evaluate the cognitive impairment of patients [130].

## 9. Conclusions

Between the first description of the disease and now, much has become clear in terms of the disease pathogenesis and clinical and laboratory diagnosis, and many approaches have been developed and tested for treatment of the disease. However, several issues remain open and need to be solved.

The disease pathogenesis, for many years, only considered the result of undegraded GAG accumulation in the different organs, is now known to be more complex, with further unsuspected aspects, often shared with other LSDs. A deep comprehension of these pathogenic mechanisms may hopefully permit the identification of other, more specific therapeutic targets that can then be evaluated to obtain normalization of the pathogenic scenario. This especially applies to the neuropathogenesis, considering that Hunter patients exhibiting different degrees of CNS involvement may be identified within the same family. Therefore, epigenetic factors will also likely need to be investigated. 

From a therapeutic point of view, 14 years after the introduction of the presently applied ERT protocol, it is probably time to reconsider it, by evaluating different dosages and/or frequencies of administration. Furthermore, it is important to redefine guidelines for ERT patients’ enrolment or discontinuation. As for brain treatment, the main problem to be solved remains, which is to find a safe, efficient, and effective therapeutic approach, together with the set-up of a poorly invasive procedure.

Hopefully, all these efforts will help Hunter patients and their families to face all the daily difficulties that they encounter when living together and fighting with such a complex and life-threatening disease.

## Figures and Tables

**Table 1:** Frequency of the different types of variants reported for the *IDS* gene. Total number of variants *n* = 658 (source: HGMD professional 2019.1).

Type of Variants	Frequency (%)
Missense/nonsense variants	49.8
Small deletions	19.0
Splicing variants	9.2
Gross deletions	8.2
Small insertions	7.9
Complex rearrangements	3.0
Small indels	2.3
Gross insertions	0.6

**Table 2:** Animal models for Mucopolysaccharidosis type II. HR: homologous recombination.

Year of Publication	Animal Model	Model Generation	Reference
1998	Dog	Spontaneous (genetic analysis not available)	[220]
1999	Mouse	Knock-out: substitution of exon 4 and part of exon 5 by HR with the neomycin resistance gene	[19,218]
2010	Mouse	Knock-out: substitution of 1485 bp of exon 2 and exon 3 by HR with the neomycin resistance gene	[165]
2010	Zebrafish	Knock-down by antisense morpholino oligo against the ATG translation initiation site	[221]
2012	Mouse	Knock-out: deletion from exon 2 to exon 5 (JCR Pharmaceuticals Co.)	[222]
2016	Mouse	Knock-out: deletion from exon 2 to exon 5 (Taconic Biosciences #TF1838)	[166]
2018	Zebrafish	Knock-out: deletion of 5 bp in the exon 2 by CRISPR/Cas9 approach	[200]

## Abbreviations

6MWT	six-minute walk test
DS	dermatan sulphate
ERT	enzyme replacement therapy
GAG	glycosaminoglycan
HS	heparan sulphate
HSCT	hematopoietic stem cell transplantation
IDDD	intrathecal drug delivery device
LSD	lysosomal storage disorder
MPS	mucopolysaccharidosis
NBS	newborn screening
NSC	neural stem cell
uGAG	urinary glycosaminoglycan
